# The effect of contouring on fatigue resistance of three types of fracture fixation plates

**DOI:** 10.1186/s13018-016-0439-1

**Published:** 2016-09-26

**Authors:** Angela S. P. Lin, Chelsea M. Fechter, Mark Magill, Felix Wipf, Thomas Moore, Robert E. Guldberg

**Affiliations:** 1 George W. Woodruff School of Mechanical Engineering, Georgia Institute of Technology, 801 Ferst Dr., Atlanta, GA 30332 USA; 2 Parker H. Petit Institute for Bioengineering & Bioscience, Georgia Institute of Technology, 315 Ferst Dr. NW, Atlanta, GA 30332 USA; 3 Wallace H. Coulter Department of Biomedical Engineering, Georgia Institute of Technology, 313 Ferst Dr., Atlanta, GA 30332 USA; 4 Department of Orthopaedics—Trauma, Emory University School of Medicine, 80 Jesse Hill Jr. Dr. SE, Atlanta, GA 30303 USA; 5 Stryker Trauma AG, Dr. Homer Stryker Str. 1, 2545 Selzach, Switzerland

**Keywords:** Bone plates, Bone fractures, Fracture fixation

## Abstract

**Background:**

Metallic reconstruction plates used for fracture stabilization typically require intraoperative contouring for patient-specific anatomical fit. Despite this, characterization of plate mechanical properties after contouring has previously been limited.

The objective of this study was to assess whether contouring affects fatigue resistance for three types of Stryker seven-hole stainless steel (SS) 316LVM fracture fixation plates. The hypothesis was that for each plate type, more contouring repetitions would result in lower fatigue resistance.

**Methods:**

Plates were contoured using a bench-top plate bender to ±20° either 0×, 3×, 6×, or 9× (*n* = 5 per group) and tested in the straight configuration. Cyclic four-point bending was applied in an incremental stepwise staircase approach (one step = 100,000 cycles, 10 Hz) until failure (defined as brittle fracture or plastic deformation of 10° permanent bend). Moment-cycle product (MCP) was computed as the summation of maximum moment × number of cycles and used as the primary measure of fatigue resistance.

**Results:**

No significant differences in fatigue resistance were detected between contouring groups for Basic Fragment Set (BFS) Reconstruction Plates. Significantly lower fatigue resistance was measured for 9× contoured Matta Pelvic System (MPS) Straight Plates compared to 0× contoured plates (*p* = 0.023). MPS Flex Plates contoured 3× had greater fatigue resistance than 0× contoured (*p* = 0.031) and 9× contoured plates (*p* = 0.032).

**Conclusions:**

This work provides fatigue resistance-based evidence that clinicians should avoid high repetitions of contouring for MPS Straight Plates. Meanwhile, BFS Reconstruction Plates and MPS Flex Plates are not negatively affected by contouring. These results allow for improved intraoperative decisions about using or discarding plates after multiple contouring repetitions.

## Background

Pelvic fractures are challenging to operatively stabilize. Fractures typically require indirect reduction without visualization of the fracture, and reconstruction plates are often placed under large muscle groups and neurovascular bundles that further inhibit visibility. High variance in individual pelvic sizes, shapes, and complex fracture patterns make pre-contoured plates impractical. Plates have an initial straight or curved form and are malleable to allow contouring in multiple planes to match individual patient anatomy for internal fixation. Several contouring repetitions are often necessary to achieve adequate bony contact across the entire plate, particularly for inexperienced surgeons and low fracture visibility. While there have been fears that multiple contouring may cause decreased fatigue resistance resulting in loss of fracture reduction and plate failure with functional loading, quantification of the effects of multiple contouring repetitions on plate mechanical properties has not previously been performed.

Fatigue represents the accumulation of damage incurred by a material when it is subjected to repetitive cyclic loading at stress magnitudes lower than that required to cause failure in a single cycle [1, 2]. Fracture due to fatigue develops through the initiation of microscopic cracks followed by gradual propagation through the implant. The study of fatigue properties for medical device design is clearly of fundamental importance [3, 4]—particularly for orthopedic devices implanted in weight-bearing fracture repair sites [5–13]. For plates used for fracture fixation, clinically relevant fatigue conditions would be most consistent with immediate post-operative low-impact, high-repetition exercises and not unexpected falls or acute instances of extreme loading.

Static four-point bending test methods from the American Society for Testing and Materials (ASTM) standard F382-99 have been utilized to compare non-contoured plate properties for various types of long bone fixation plates [14]. The ASTM standard F382-99 specifies test methods to assess mechanical characteristics important to the in vivo performance of bone plates [15]. F382-99 has been recognized by the Food and Drug Administration (FDA) as a standard that applies to metallic bone plates for orthopedic use and affects several medical device regulation processes (including 510(k), PMA, IDE; Recognition Number 11-240) [16]. A recent study on distal tibial fixation plates indicated via finite element (FE) predictions of stress distributions validated with static four-point bending tests that Ti-6Al-4V alloy plates should not be used where large deformation contouring would be required while 316-L stainless steel plates would maintain mechanical integrity after large deformations [17]. However, effects of contouring on cyclic fatigue properties have not been previously investigated.

The goal of this study was to determine whether fatigue properties of commonly utilized stainless steel (SS) fixation plates for weight-bearing regions would be negatively affected by multiple repetitions of contouring and recontouring. Specifically, for each of three SS 316LVM plate types (Stryker Plating System Basic Fragment Set Reconstruction Plates, Matta Pelvic System Straight Plates, and Matta Pelvic System Flex Plates), fatigue resistance was quantified and compared after 0×, 3×, 6×, or 9× contouring repetitions. We hypothesized that for all plate types, contouring with a standard bench-top plate bender would decrease fatigue resistance as a function of the number of repetitions.

## Methods

### Plates and contouring

**Fig. 1:**
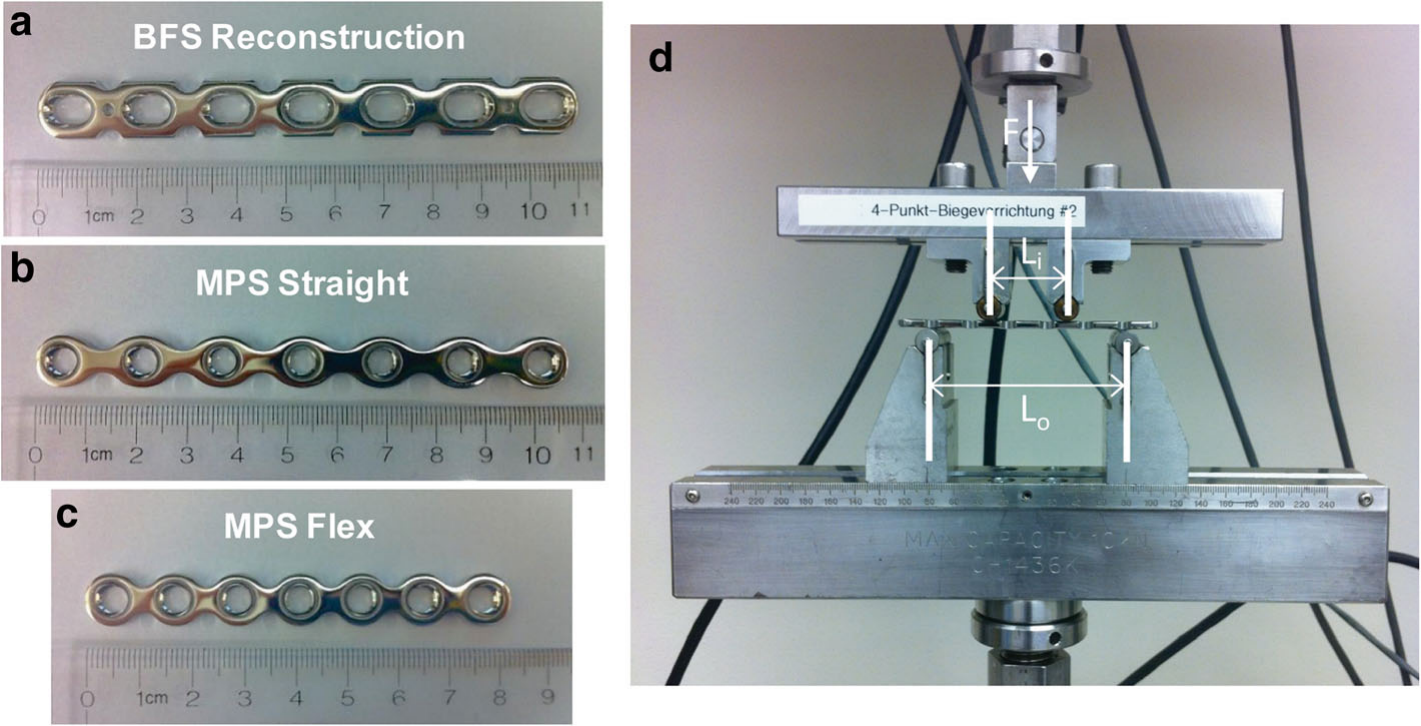
Representative pictures of (**a**) BFS Reconstruction, (**b**) MPS Straight, and (**c**) MPS Flex Plates with no contouring and before fatigue testing; (**d**) four-point bending test setup on MTS 858 Mini Bionix II (cyclic loads applied as shown, F; inner and outer span lengths L_i_ and L_o_, respectively)

Three types of seven-hole SS Stryker plates were used (provided by Stryker Trauma AG, Selzach, Switzerland, Fig. 1): Stryker Plating System (SPS) Basic Fragment Set (BFS) Reconstruction Plate (REF 432207, SS 316LVM annealed, length = 110 mm, thickness = 3.1 mm, holes for 4.5-mm screws, Fig. 1a), Matta Pelvic System (MPS) Straight Plate (REF 425707, SS 316LVM non-annealed, length = 106.5 mm, thickness = 2.5 mm, holes for 4.5-mm screws, Fig. 1b), and MPS Flex Plate (REF 425757, SS 316LVM annealed, length = 82.5 mm, thickness = 2.5 mm, holes for 4.5-mm screws, Fig. 1c). Plate dimensions and characteristics are included in Table 1. Plates were contoured 0×, 3×, 6×, or 9× (*n* = 5 for each group). Contouring was performed directly over the middle hole with a standard bench-top plate bender to +20° then −20° as 1× repetition. After the final repetition, the plate was returned to the 0° straight configuration.

**Table 1:** Plate characteristics, cyclic four-point bending span widths, cyclic fatigue initial maximum load levels, and step increments

Plate type	Annealed?	Plate length/thickness/hole spacing (mm)	Span *L*_o_ (mm)	Span *L*_i_ (mm)	Start *F*_max_ (N)/% of *M*_y_	Load step increment (N)
BFS Reconstruction	Y	110/3.1/16	80	32	300/240	12.5
MPS Straight	N	106.5/2.5/16	80	32	200/160	12.5
MPS Flex	Y	82.5/2.5/12	60	24	153/90	17

*L*_*o*_ outer span width, *L*_*i*_ inner span width for four-point bending test fixture, *M*_*y*_ bending moment at yield point (0.2 % offset method)

### Preliminary four-point bending tests

ASTM F382-99(Reapproved 2008)ε1, Standard Specification and Test Method for Metallic Bone Plates and superceding active standard F382-14 were used as guidelines for apparatus setup and fatigue test performance [15]. Plates were oriented within the test fixtures with the bone interface (concave) side facing upwards (Fig. 1d). For BFS Reconstruction Plates and MPS Straight Plates, outer (*L*_o_) and inner (*L*_i_) span widths were 80 and 32 mm, respectively; for MPS Flex Plates, span widths were *L*_o_ = 60 mm and *L*_i_ = 24 mm (Table 1). Span widths were centered about the middle hole of each plate. The lower test fixture was held stationary while the upper fixture was programmed to apply force/displacement changes. One control non-contoured plate of each type was used for preliminary static four-point bending (MTS 858 Mini Bionix II, Fig. 1d) at a constant displacement control rate of 0.1 mm/s to determine bending moment at yield (*M*_y_) using the 0.2 % yield offset method. For subsequent stepwise cyclic loading, step increments of 10 % of the minimum of these three *M*_y_ values were used, and these 10 % increments are shown, translated to the equivalent force increments, in Table 1.

### Cyclic fatigue testing (four-point bending)

**Fig. 2:**
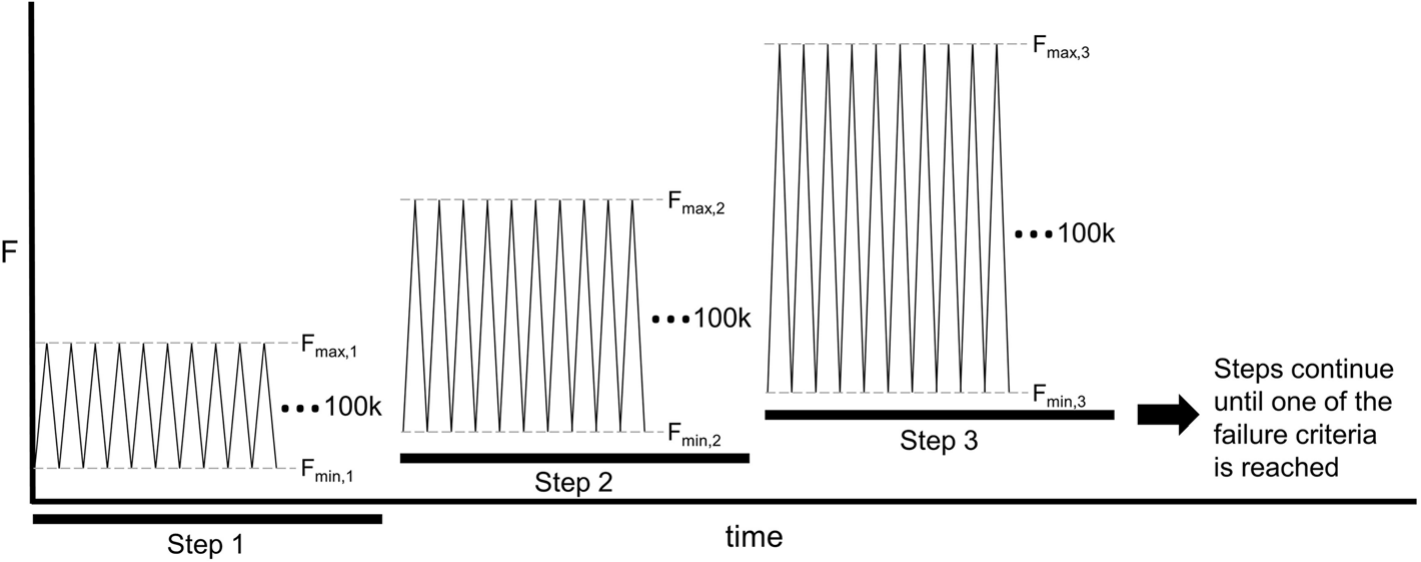
Each step consisted of 100,000 cycles at 10 Hz in the four-point bending configuration. Upper and lower load level targets for each cycle were *F*_max_ and *F*_min_, respectively, where *F*_min_ = 10 % *F*_max_ for each step. *F*_max_ was increased from one step to the next by a constant load increment corresponding to 10 % *M*_y,min_, determined in preliminary tests to failure for each plate type. These *F*_max_ load increase increments differed for the plate types due to geometry differences and are listed in Table 1. For example, for BFS Reconstruction Plates, *F*_max,*b*+1_ − *F*_max,*b*_ = 12.5 N, where *b* = step number. Tests continued with subsequent steps of incrementally increased *F*_max_ until conditions of failure were met (plate fracture or plastic deformation beyond 10° bend of the plate at 10-N load, applied between steps)

Plates were subjected to cyclic loads in a stepwise staircase approach [12]. At each step, 100,000 cycles in four-point bending were applied at 10 Hz between 10 and 100 % of the maximum load designated for that step. Figure 2 shows an illustrative load vs. time plot describing the cyclic test procedure. Load corresponding to 10 % of the pre-determined minimum *M*_y_ was used as the incremental increase of *F*_max_ between steps (12.5 N for BFS Reconstruction and MPS Straight Plates; 17 N for MPS Flex Plates, Table 1). To estimate failure load levels, preliminary cyclic fatigue testing was performed on one control plate of each type beginning at 20 % of the load corresponding to *M*_y_ and increasing stepwise as described (Fig. 2) until failure occurred. Based on these preliminary tests, subsequent plate cyclic testing began at initial *F*_max_ levels such that failure would most likely be reached within eight steps (~24 h). See Table 1 and Fig. 2 for step details.

### Failure criteria

Failure was defined as brittle plate fracture due to cyclic loading or plastic deformation failure resulting in 10° permanent bending of the plate (empirically determined to be equivalent to 1.95-mm crosshead displacement) at low load (10 N). Between each 100,000-cycle step, the plate was loaded to 10 N, and crosshead displacement was measured to determine whether the deformation criterion was reached.

### MCP as an assessment of cyclic fatigue resistance

**Fig. 3:**
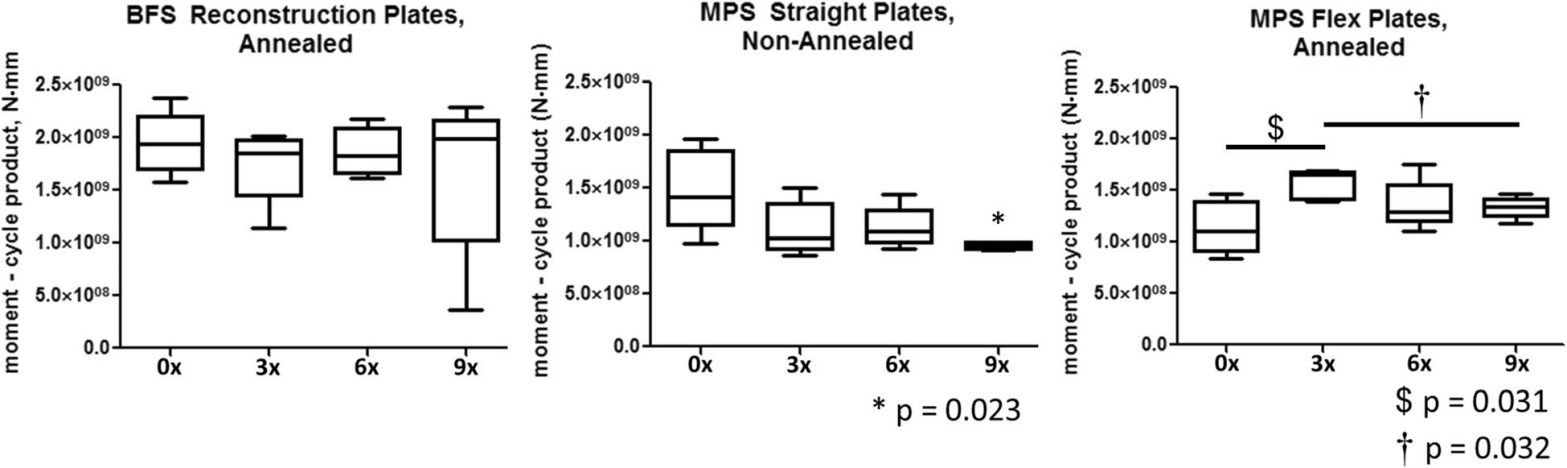
*Boxplots* depict median moment-cycle product (MCP) values, 1st and 3rd quartiles, and *whiskers* indicate maximum and minimum values (*n* = 5 for each group). **a** BFS Reconstruction Plates: no significant differences in MCP between groups. **b** MPS Straight Plates: plates contoured 9× exhibited significantly lower MCP to failure than those contoured 0× (*p = 0.023). **c** MPS Flex Plates: 3× contoured plates exhibited significantly higher MCP to failure than 0× (^$^ *p* = 0.031) and 9× (^†^ *p* = 0.032)

For each step endured until failure, the maximum moment produced during that step was multiplied by the number of cycles in that step, and these values were summed to compute a total moment-cycle product (MCP) to failure. This parameter was used as the primary measure of cyclic fatigue resistance, similar to the moment-cycle integral metric used in Schmidt et al. for testing locking fixation plates [12], as it included considerations for geometry and total cycles endured whereas other fatigue property measures did not represent the combination of these factors as completely. Boxplots were generated to depict MCP median values, 1st and 3rd quartiles, and minimum and maximum values (Fig. 3).

### Additional cyclic fatigue properties

The number of plates that failed according to each failure criterion (brittle or plastic) for each group and the average cycles to failure, average failure load level (*F*_fail_), and average failure moment (*M*_fail_) are reported in Table 2.

**Table 2:** Cyclic fatigue data for plate types and groups

Plate type	Group	Brittle fracture failure	Plastic def. failure	Avg cycles to failure	Avg *F*_fail_ (N)	Avg *M*_fail_ (N · mm)	Avg MCP (N · mm)
BFS Reconstruction	0×	4/5	1/5	497600 ± 30535	358 ± 3.1	4290 ± 37	1.94 × 10^9^ ± 1.31 × 10^8^
	3×	2/5	3/5	448800 ± 37803	350 ± 4.0	4200 ± 47	1.74 × 10^9^ ± 1.56 × 10^8^
	6×	2/5	3/5	478000 ± 24160	355 ± 3.1	4260 ± 37	1.86 × 10^9^ ± 1.03 × 10^8^
	9×	3/5	2/5	429200 ± 85320	348 ± 12.1	4170 ± 145	1.67 × 10^9^ ± 3.42 × 10^8^
MPS Straight	0×	5/5	0/5	538200 ± 55203	260 ± 7.3	3120 ± 87	1.48 × 10^9^ ± 1.73 × 10^8^
	3×	5/5	0/5	425400 ± 37214	245 ± 5.2	2942 ± 62	1.11 × 10^9^ ± 1.11 × 10^8^
	6×	5/5	0/5	423200 ± 28798	248 ± 4.7	2970 ± 56	1.12 × 10^9^ ± 8.58 × 10^7^
	9×	5/5	0/5	364600 ± 5741	238 ± 0	2850 ± 0	9.49 × 10^8^ ± 1.64 × 10^7^
MPS Flex	0×	0/5	5/5	632000 ± 50465	252 ± 9.9	2264 ± 89	1.14 × 10^9^ ± 1.15 × 10^8^
	3×	3/5	2/5	812000 ± 25938	282 ± 4.2	2540 ± 37	1.56 × 10^9^ ± 6.55 × 10^7^
	6×	1/5	4/5	726600 ± 43271	272 ± 5.4	2448 ± 48	1.36 × 10^9^ ± 1.07 × 10^8^
	9×	5/5	0/5	717200 ± 18985	269 ± 7.6	2417 ± 31	1.33 × 10^9^ ± 4.54 × 10^7^

Number of plates that failed due to either brittle fracture or plastic deformation are indicated (total *n* = 5 per group). Average cycles to failure, failure load level (*F*_fail_), failure moment (*M*_fail_), and moment-cycle product (MCP) are shown as mean ± standard error

### Statistics

Within each plate type, mean MCP was compared via Kruskal-Wallis non-parametric one-way analysis of variance. Where differences in mean MCP due to contouring were detected (*α* = 0.1), comparisons between repetition groups were performed with two-tailed Mann-Whitney *U* tests with Monte Carlo *p* value (95 % confidence interval, 10,000 samples) in SPSS V20 (IBM, New York, USA). Post hoc power and sensitivity analyses were performed for one comparison (MPS Straight Plate 0× vs 3× groups) using the following parameters: test family—*t* tests, difference in means (Wilcoxon-Mann-Whitney test, two groups), two-tailed, *α* = 0.05, power = 0.8 (G*Power 3.1.9.2, Heinrich-Heine-Universität, Düsseldorf, Germany) [18, 19].

## Results

### Effects of contouring on BFS Reconstruction Plates (432207)

There was no significant effect of contouring on MCP for BFS Reconstruction Plates (Fig. 3a, Table 3). Additionally, there was no contouring group for the BFS Reconstruction Plates that exhibited failure due to only one mode (Table 2).

**Table 3:** Between group comparison *p* values of moment-cycle product (MCP) within each plate type

Plate type	Group	0×	3×	6×	9×
BFS Reconstruction	0×	–	>0.5	>0.5	>0.5
	3×	>0.5	–	>0.5	>0.5
	6×	>0.5	>0.5	–	>0.5
	9×	>0.5	>0.5	>0.5	–
MPS Straight	0×	–	0.156	0.219	0.023*
	3×	0.156	–	>0.5	0.322
	6×	0.219	>0.5	–	0.051
	9×	0.023*	0.322	0.051	–
MPS Flex	0×	–	0.031^$^	0.344	0.301
	3×	0.031^$^	–	0.145	0.032^†^
	6×	0.344	0.145	–	>0.5
	9×	0.301	0.032^†^	>0.5	–

Symbols correspond to Fig. 1 significance markers (*p = 0.023 for MPS Straight Plates between 0× and 9× contour repetitions; ^$^ *p* = 0.031 for MPS Flex Plates between 0× and 3× contour repetitions; ^†^ *p* = 0.032 for MPS Flex Plates between 3× and 9× contour repetitions)

### Effects of contouring on MPS Straight Plates (425707)

Contouring produced a significant effect on MCP of MPS Straight Plates. Plates contoured 9× required significantly lower MCP to fail than control plates contoured 0× (*p* = 0.023) (Fig. 3b, Table 3), indicating that nine repetitions of contouring produced negative effects on plate fatigue resistance. Plates from all contouring groups failed due to brittle fracture (Table 2).

### Effects of contouring on MPS Flex Plates (425757)

There was a significant effect of contouring on MCP for MPS Flex Plates. Plates contoured 3× exhibited higher MCP compared to control plates contoured 0× (*p* = 0.031) and plates contoured 9× (*p* = 0.032) (Fig. 3c, Table 3). These data demonstrated no negative effects of contouring on fatigue resistance and plate strengthening after three contouring repetitions. Plates in the control 0× contoured group all failed due to plastic deformation while plates in the 9× contoured group all failed due to brittle fracture (Table 2).

## Discussion

The goal of this work was to assess the fatigue properties of three types of stainless steel fixation plates used for fracture stabilization after contouring. Surgeons have not previously had guidelines for accessing the potential negative clinical sequelae of the common practice of multiple contouring on the mechanical integrity of reconstruction plates. Our hypothesis was that the ability of all plates to resist fatigue would decrease with increasing number of contouring repetitions. Contrary to our hypothesis, only one plate type (MPS Flex Plates) experienced significantly decreased fatigue resistance with increased number of contour repetitions and only at the highest number (9×) of repetitions.

This study included a few limitations. Although the plate bender used in this study was identical to those used clinically for single-plane contouring, plates may further undergo multi-planar manual contouring before they are implanted. Therefore, more complex clinically equivalent post-contouring states may not have been achieved. Despite this, the contouring protocol was considered aggressive as it involved large (±20°) deformations in a localized position at the center of the plates. Additionally, plates underwent high-frequency and high-volume cycling, and average cyclic failure loads were above 80 % of initially determined yield loads for each plate type. The study was also limited in scope to fatigue properties, which are consistent with cyclic post-operative mobilization protocols but do not represent the ability of plates to withstand acute loading conditions such as an accidental fall.

Results were largely contrary to the original study hypothesis. There were no deleterious effects of high contouring repetitions on fatigue resistance for BFS Reconstruction Plates or MPS Flex Plates. Surprisingly, an increase in the ability of MPS Flex Plates to withstand cyclic fatigue loading after 3× contouring was seen. This may be attributed to a strengthening of the metal, through repeated plastic deformation, with lower numbers of contours. MPS Straight Plates contoured 9× experienced lower MCP prior to failure, indicating a significantly detrimental effect of contouring. This may be attributed to strain hardening resulting in a loss of ductility. Combined with observations that all MPS Straight Plates (the only non-annealed plate type) failed via brittle fracture, this study suggests that nine or more contouring repetitions should be avoided in the clinical setting for these plates. Though the study was not designed to explicitly assess effects of annealing, it is possible that this thermal material treatment process produced microstructural changes, resulting in a difference in the ability to retain mechanical integrity after high contouring repetitions. No statistical differences in MCP were detected between control and 3× or 6× contoured plates for the MPS Straight Plate type. A post hoc power analysis for control vs. 3× contoured MPS Straight Plates determined that power = 0.34. A sensitivity analysis at power = 0.8 resulted in effect size (ES) of 2.09 needed to detect a difference with the number of samples available per group (*n* = 5), while the actual ES determined by post hoc power analysis was 1.14. These analyses suggest that perhaps a larger sample size would have allowed detection of differences in mean MCP between control and 3× or control and 6× contoured MPS Straight Plates using interferential statistics.

## Conclusions

Intraoperative plate contouring occurs frequently in the treatment of pelvic fractures. Unlike long bone fractures where there is direct visualization of the bone during the contouring process, pelvic fractures require indirect contouring due to low visibility associated with the pelvis and surrounding musculature. Clinical solutions to avoid excess intraoperative contouring include using pre-contoured plates or pre-operatively contouring plates on a standard anatomic model. However, the considerable anatomic variance of individual pelvic anatomies makes these techniques non-ideal. Techniques utilizing CT imaging to generate 3D models for the purpose of designing patient-specific plate shapes have been developed [20–22], but these capabilities are costly, not widely available, and may be too time consuming for trauma situations. Eliminating intraoperative indirect contouring of fixation plates may not be possible. As such, this study provides clinicians with guidelines for intraoperative contouring of pelvic plates and resultant recovery progress decisions. The work suggests that clinicians should avoid high contouring repetitions of SS 316LVM Stryker MPS Straight Plates. Meanwhile, SS 316LVM Stryker BFS Reconstruction Plates and MPS Flex Plates would not be negatively affected by contouring. These results allow better intraoperative decisions to be made regarding whether plates contoured multiple times should be used or discarded. This in turn eliminates the factor of decreased plate fatigue life from the clinical decision to allow early partial weight bearing during the recovery process.

## Abbreviations

ASTM: American Society for Testing and Materials
BFS: Basic Fragment Set
ES: Effect size
FDA: Food and Drug Administration
FE: Finite element
MCP: Moment-cycle product
MPS: Matta Pelvic System
SPS: Stryker Plating System
SS: Stainless steel
